# Effect and Biocompatibility of a Cross-Linked Hyaluronic Acid and Polylactide-*co*-glycolide Microcapsule Vehicle in Intratympanic Drug Delivery for Treating Acute Acoustic Trauma

**DOI:** 10.3390/ijms22115720

**Published:** 2021-05-27

**Authors:** Jung-Ah Cho, Bong Jik Kim, Yu-Jung Hwang, Shin-Wook Woo, Tae-Soo Noh, Myung-Whan Suh

**Affiliations:** 1 Department of Otorhinolaryngology–Head and Neck Surgery, Seoul National University Hospital, Seoul 03080, Korea; whwjddk2000@hanmail.net (J.-A.C.); eliteyujung@naver.com (Y.-J.H.); book2929@naver.com (S.-W.W.); garnet1385@naver.com (T.-S.N.); 2 Department of Otolaryngology–Head and Neck Surgery, College of Medicine, Chungnam National University, Daejeon 35015, Korea; bongjik.kim@cnu.ac.kr; 3 Department of Otolaryngology–Head and Neck Surgery, Chungnam National University Sejong Hospital, Sejong 30099, Korea

**Keywords:** acute acoustic trauma, drug delivery, hyaluronic acid, microcapsule, dexamethasone, hearing loss

## Abstract

The treatment of acute hearing loss is clinically challenging due to the low efficacy of drug delivery into the inner ear. Local intratympanic administration of dexamethasone (D) and insulin-like growth factor 1 (IGF1) has been proposed for treatment, but they do not persist in the middle ear because they are typically delivered in fluid form. We developed a dual-vehicle drug delivery system consisting of cross-linked hyaluronic acid and polylactide-*co*-glycolide microcapsules. The effect and biocompatibility of the dual vehicle in delivering D and IGF1 were evaluated using an animal model of acute acoustic trauma. The dual vehicle persisted 10.9 times longer (8.7 days) in the middle ear compared with the control (standard-of-care vehicle, 0.8 days). The dual vehicle was able to sustain drug release over up to 1 to 2 months when indocyanine green was loaded as the drug. One-third of the animals experienced an inflammatory adverse reaction. However, it was transient with no sequelae, which was validated by micro CT findings, endoscopic examination, and histological assessment. Hearing restoration after acoustic trauma was satisfactory in both groups, which was further supported by comparable numbers of viable hair cells. Overall, the use of a dual vehicle for intratympanic D and IGF1 delivery may maximize the effect of drug delivery to the target organ because the residence time of the vehicle is prolonged.

## 1. Introduction

Sensorineural hearing loss (SNHL) is associated with inner ear hair cell damage and can be caused by genetic disorders, infection, loud noises, or ototoxic drugs. There is no effective treatment for chronic SNHL except cochlear implants in selected cases. For acute onset hearing loss including sudden SNHL (SSNHL), steroid treatment can be effective. Systemic steroid therapy is the most common treatment for SSNHL [1,2] and a higher concentration of steroid is associated with a better treatment outcome [3,4]. However, large doses of systemic steroid can lead to undesirable adverse effects [5]. In addition, due to the blood-labyrinth barrier, it is difficult to maintain a sufficient level of steroid in the local target organ (cochlea) [6]. Target-oriented local delivery methods such as intratympanic (IT) injection of dexamethasone (D) have been introduced as alternative methods. Due to direct absorption through the round window membrane (RWM), IT injection can achieve a higher concentration of steroid in the perilymph compared with systemic administration [7,8,9,10,11]. A systematic review showed that IT steroids can be a valuable solution for patients with acute SNHL who either cannot tolerate systemic steroid therapy or do not respond to treatment [12]. The American Academy of Otolaryngology—Head and Neck Surgery also recommended that clinicians should offer IT steroid perfusion when patients exhibit incomplete recovery from SSNHL [13].

Insulin-like growth factor 1 (IGF1) was reported to be a novel agent facilitating the protection and regeneration of cochlear hair cells. IGF1 was first isolated from human serum in the 1950s and is an important growth mediator, with a mechanism of action similar to that of insulin. Compared to insulin, IGF1 has a 50- to 100-fold higher proliferation effect [14,15]. Because of its important role in cell proliferation and differentiation, IGF1 is believed to affect the developmental process of the inner ear. One in vitro study showed that exogenous IGF1 application protected chick otic vesicles from cell death after pro-apoptotic stimuli [16]. In an acute acoustic trauma animal model, IGF1 significantly improved hearing thresholds, indicating that IGF1 can protect hair cells against noise exposure [17,18]. Clinically, the efficacy of IGF1 administration was demonstrated in acute SNHL patients who did not benefit from systemic steroid treatment [19]. IGF1 has been proposed as a novel adjuvant treatment for patients with SSNHL [20]. In this study, we combined steroid with IGF1 to ensure a positive treatment outcome. Considering that the mode of action differs between steroids (anti-inflammatory effect) and IGF1 (inhibition of hair cell apoptosis and proliferation of supporting cells), the two drugs can function synergistically in hair cell recovery [21].

The current IT treatment has a limitation in that the drug persists in the middle and inner ear for a very short time. In addition, the fluid form of the drug is easily drained through the Eustachian tube. It has been reported that a pharmacokinetically meaningful concentration of D in the perilymph can be sustained for 12 h with a mean residence time of 3.6 h [22]. Thus, the current standard-of-care method requires frequent IT injections, which is associated with a high medical expense and increased risk of procedure-related complications. If the drug is loaded in a vehicle that persists longer in the middle ear, more drug could be delivered into the inner ear, which in turn can result in better audiological outcomes. In our previous study, conventional hyaluronic acid (HA) was a highly biocompatible IT vehicle [23]. However, it did not guarantee sufficient residence time in the middle ear [23]. Because oral steroid medication is prescribed for 1–2 weeks for patients with acute SNHL, it would be ideal if the vehicle could last for 1–2 weeks in the middle ear and elute the drug during this period. To address the shortcomings of current IT treatment, we designed a dual vehicle using cross-linked HA and polylactide-*co*-glycolide (PLGA) microcapsules [24,25]. By increasing the residence time and achieving sustained drug release, we aimed to enhance treatment outcomes. In this study, we compared the treatment effect and biocompatibility between the dual vehicle (cross-linked HA and PLGA microcapsule) and standard-of-care vehicle (saline). The drugs (D and IGF1) were the same in both groups. Specifically, we focused on the residence time of the vehicle, endoscopic and histological adverse inflammatory reactions, changes in the hearing threshold, and number of hair cells surviving after acute acoustic trauma.

## 2. Results

### 2.1. Residence Time of the Drug/Vehicle as Evaluated by Micro CT

**Figure 1:**
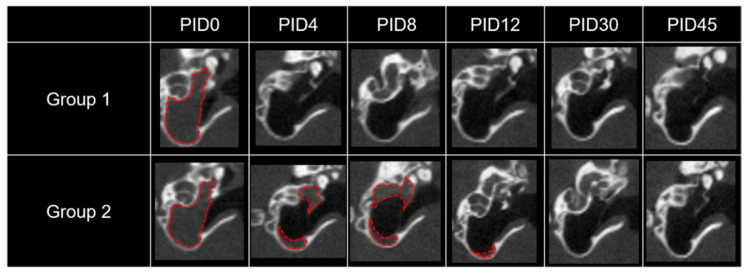
Micro CT of the drug/vehicle in the middle ear. Signal attenuation (indicated by red dotted lines) in the middle ear indicates the mixture of drugs and vehicle. Based on micro CT evaluation from PID0 to 45, the drug/vehicle lasted in the middle ear for less than 1 day in group 1 and for 8 to 12 days in group 2.

As shown in Figure 1, soft tissue densities (signal attenuation according to drug/vehicle) in the bulla were clearly identified by micro computed tomography (CT) after IT drug delivery. The drug/vehicle completely filled the middle ear on the day of IT injection (post injection day (PID)0) in both groups. However, 4 days later, no attenuation was found in group 1 (D and IGF1 in saline), whereas some drug/vehicle was still visible in group 2 (microcapsulated D (mD) and IGF1 loaded in a cross-linked HA vehicle). Quantitatively, the drug/vehicle lasted for 0.8 ± 0.1 days in group 1 and 8.7 ± 4.7 days in group 2. The residence time was significantly longer in group 2 compared to group 1 (*p* = 0.041, Mann-Whitney *U* test).

### 2.2. Visible Light Endoscopy

**Figure 2:**
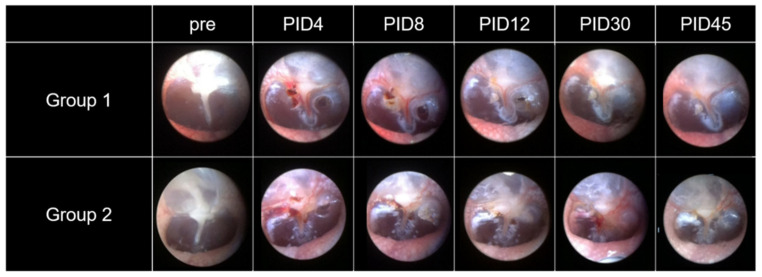
Visible light endoscopy of tympanic membrane perforations. Perforations that were generated during the injection procedure healed well in both groups. It took 13.2 ± 2.4 days for the perforation to heal completely in group 1 and 11.6 ± 4.0 days in group 2.

Serial changes in the tympanic membrane (TM) after IT treatment are shown in Figure 2. It took 13.2 ± 2.4 days for the TM perforation to heal completely in group 1 (n = 13). In group 2, it took 11.6 ± 4.0 days. There was no significant difference in the time for complete healing of the TM between the two groups (*p* = 0.194, Mann-Whitney *U* test). The incidence of inflammatory adverse reactions was 23% (3/13) in group 1 and 36% (4/11) in group 2. There was no significant difference between the two groups (*p* = 0.659, Fisher’s exact test). Typical endoscopic findings indicating inflammatory adverse reactions in group 2 are described (Supplementary Figure S1).

### 2.3. Real-Time Fluorescence Endoscopy with Indocyanine Green (ICG)

**Figure 3:**
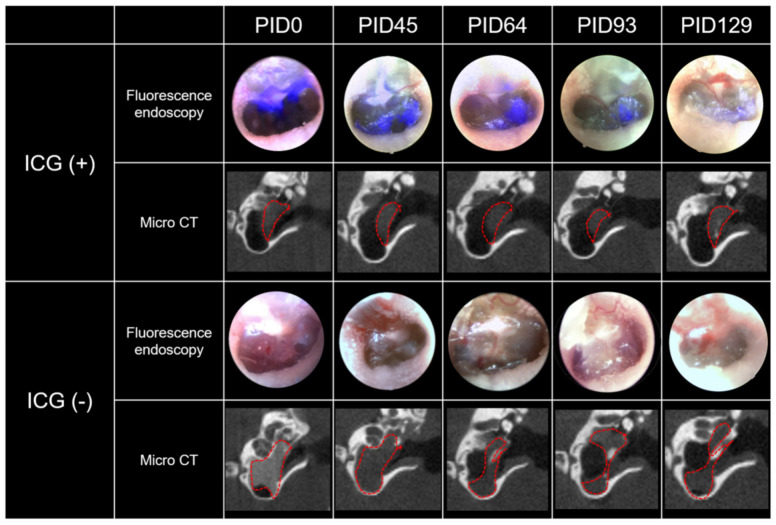
Real-time fluorescence endoscopy of ICG and micro CT in the same ears. The ICG (+) group was injected with 5 mg/mL of ICG in cross-linked HA, whereas the ICG (−) group was injected with a control dye (iodinated dye with no fluorescence) in cross-linked HA. The fluorescence was very strong on PID0 in the ICG (+) group. This fluorescence was clearly visible up to PID64 to 93 and became very faint on PID129. Meanwhile, no fluorescence was observed in the ICG (−) group. This pattern of fluorescence was independent of the vehicle because a large amount of vehicle was always visible in the middle ear according to micro CT observations.

In the ICG (+) ears, a strong fluorescent signal was observed in the TM on the day of injection (PID0). This fluorescent signal started to decrease with time, and on the last day of the experiment (PID129), the signal was barely visible (Figure 3). A clear signal was identified up to 64 to 93 days. On the contrary, no fluorescence signal was identified from the first day of injection (PID0) to the last day (PID129) in the ICG (−) group. This finding was consistent in all ICG (+) and ICG (−) ears. Due to the cross-linked HA with a high degree of bridging (1/250–1/1000), the vehicle was always present in the middle ear throughout the whole period in both groups, which was supported by micro CT (Figure 3).

### 2.4. Histologic Findings of the TM and Middle Ear Mucosa

Histologically, there was no difference in the TM and mucosa at the base of the bulla between groups 1 and 2 (Supplementary Figure S2). Quantitatively, the mean thickness of the TM was 12.4 ± 6.6 µm in group 1 and 13.3 ± 7.4 µm in group 2, with no significant difference observed (*p* = 0.571, Mann-Whitney *U* test test). The thickness of the mucosa at the base of the bulla was 22.3 ± 3.6 µm in group 1 and 24 ± 6.5 µm in group 2, with no significant difference observed (*p* = 0.974, Mann-Whitney *U* test test).

### 2.5. Audiometric Assessment Using the Auditory Brainstem Response (ABR)

**Figure 4:**
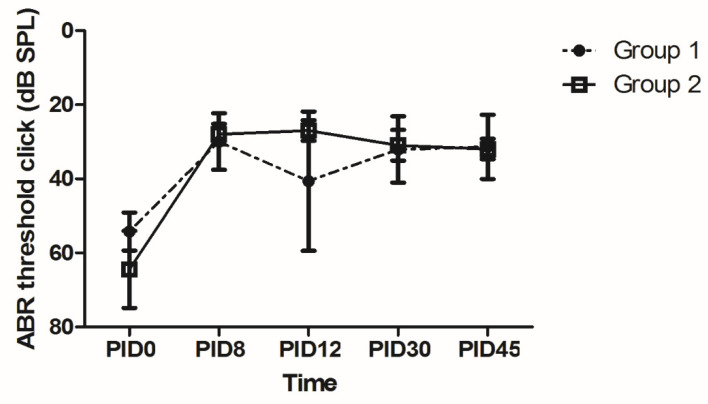
Audiometric assessment based on the auditory brainstem response (ABR). The hearing threshold significantly recovered on PID8. The hearing in both groups was comparably good after 30 to 45 days. The final hearing threshold was 31.4 ± 3.3 dB SPL and 32 ± 1.2 dB SPL in groups 1 and 2, respectively.

The increased hearing threshold after acute acoustic trauma recovered on PID8 in both groups. The hearing threshold on PID8 was significantly better than that measured on PID0 (*p* = 0.017 and 0.043 for groups 1 and 2, respectively, Wilcoxon signed rank test). After 45 days, the hearing threshold was 31.4 ± 3.3 dB SPL and 32 ± 1.2 dB SPL in groups 1 and 2, respectively. No difference was observed in the ABR threshold between the two groups (Figure 4).

### 2.6. Hair Cell Count

**Figure 5:**
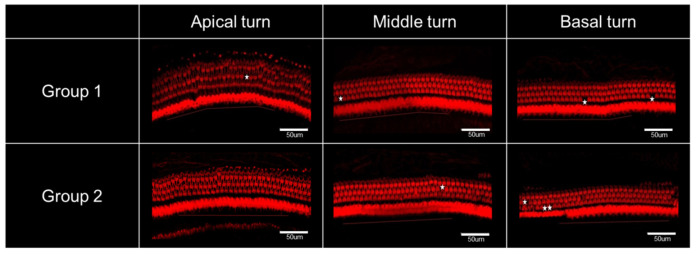
Confocal microscopy of cochlear hair cells. The organ of Corti appeared normal in both groups with minimal hair cell loss (white asterisks) in all three turns of the cochlea. When the number of hair cells was compared, there was no difference between groups 1 and 2.

The organ of Corti appeared almost normal with minimal hair cell loss in both groups (Figure 5). The numbers of outer hair cells (OHCs) in the basal, middle, and apical turns and inner hair cells (IHCs) in all three turns were counted (Table 1). When the numbers of hair cells were compared between groups 1 and 2, no differences were observed.

## 3. Discussion

Based on our results, the drug/vehicle lasted significantly longer when the dual vehicle was used. That is, the drug/vehicle was visible in the middle ear by micro CT for 8.7 days in group 2, whereas it was only visible for 0.8 days in group 1 (10.9-fold longer residence time). This result is superior to that of conventional HA. In our previous study, we used conventional non-cross-linked HA as a vehicle, and observed an unsatisfactory residence time of 1.8 ± 2.4 days [23]. Although the basic material (HA) is the same, cross-linking the HA and adding

PLGA microcapsules increased the residence time from 1.8 days to 8.7 days (4.8-fold longer). It should be noted that the residence time can be tailored by adjusting the degree of bridging in cross-linked HA. When the degree of bridging was increased from 1/10,000 to 1/250–1/500, the residence time of the vehicle was extended from 8.7 days to 129 days. Our results demonstrate that the dual vehicle can significantly extend the residence time of the drug/vehicle in the middle ear. These results are in agreement with those seen for other IT vehicles, such as thermosensitive gels, liposomes, film forming agents, and superparamagnetic nanoparticles, which can extend drug residence time to several days to months [26,27,28,29].

It may be difficult to differentiate between the drug and vehicle based on CT attenuation. That is, the CT signal may be attenuated by either a vehicle loaded with drug or an empty vehicle with no drug. A vehicle that lasts for 8.7 days in the middle ear may not be useful if the drug is released entirely within several hours. To evaluate the residence time of the drug independent of the vehicle, we performed an additional experiment with real-time fluorescence endoscopy. The ICG fluorescence was clearly visible for 64 to 93 days, implying that the cross-linked HA can serve as a depot and elute the drug over 2 to 3 months. The chemical and biological properties of D and ICG are different and we cannot directly apply the ICG results to D. However, there is no ideal method to visualize D without changing its original molecular properties and size. Currently, fluorescence endoscopy of ICG may be a good alternative method for visualizing the residence time of a drug independent of the vehicle. Considering the long residence time of ICG, the residence time of D in the middle ear can also be prolonged using the dual vehicle. Although not measured in the middle ear, former studies reported that micro-encapsulation could sustain the release time of D for up to 30 days in the inner ear [29]. The treatment outcome was satisfactory in both groups. That is, the hearing threshold was close to normal (30 dB SPL) and hair cell loss was minimal in both groups. Based on our previous study, the hearing threshold was reported to be 42.5 dB SPL on PID45 when no treatment was applied [23]. Considering that the hearing threshold in this study was 31.4 ± 3.3 dB SPL (group 1) and 32 ± 1.2 dB SPL (group 2) on PID45, D and IGF1 provided a 10-dB gain regardless of the vehicle. The number of hair cells was also greater with our treatment (73.0–73.1 cells/200 µm in group 1 and 71.6–73.6 cells/200 µm in group 2) compared to the previously reported outcome (64.1 cells/200 µm) with no treatment [23]. The treatment outcome exhibited a ceiling effect and did not differ between groups 1 and 2. The sustained release system employed in this study was hypothesized to achieve a more effective treatment outcome by extending the time of drug delivery. Theoretically, cross-linked HA would sustain the drug effect by prolonging its residence time. Microcapsules would further slow the release of D, maintaining a high concentration of D for a longer time [30]. The longer residence time would increase the chances of the drug contacting the RWM and diffusing into the inner ear [31]. We plan to assess a different animal model with a more severe degree of permanent hearing loss in the future. If the hearing loss is more severe, the long-lasting drug/vehicle used in group 2 may result in a better treatment outcome due to the sustained effect of D and IGF1.

In this study, approximately one-third of the treated ears exhibited an adverse inflammatory reaction. To be used as a clinically useful drug/vehicle, biocompatibility issues and effectiveness should be considered. The causes of the adverse inflammatory reactions remain unclear. However, the IT injection procedure (23% in group 1) and the vehicle (cross-linked HA and PLGA microcapsules; 36% in group 2) may be associated with this result. HA is very biocompatible and has been studied in various parts of the body, with the United States Food and Drug Administration having approved its use for eye surgery and joint pain relief [32]. However, the byproduct of covalent bonding between Tet and TCO can be inflammatory. PLGA is also biocompatible [33], but it has not been studied thoroughly in the ear. The TM is very sensitive to inflammation and infection, requiring extra biocompatibility. For example, methoxy-polyethylene glycol-b-polycaprolactone block copolymer has been reported to induce significant inflammatory adverse reactions when injected in the middle ear [23], although it is not known to cause adverse reactions in soft tissues [34]. However, it should be noted that the inflammation found in this study was transient and not severe. The endoscopic findings returned to normal within 20 to 60 days (Supplementary Figure S1). Histologic findings on PID60 did not differ between the two groups. One-third of the treated ears appeared to have experienced an adverse inflammatory reaction, but no macroscopic (TM endoscopy) or microscopic (histology of the middle ear mucosa) sequelae were detected after 2 months. There are several limitations in this study. First, the prolonged residence time due to the vehicle may not accurately determine treatment outcome. The effect of IT drug delivery is associated with middle ear residence time because the drugs pass through the RWM via passive diffusion [31]. However, hearing outcomes are not always correlated with residence time. In a recent animal study using phospholipid-based nanoparticles for inner ear drug delivery, D loaded in nanoparticles improved hearing at all tested frequencies [35]. It should be noted that the hearing was assessed only once (at 7 days after ototoxic hearing loss) and the long-term effect remains unknown [35]. Other studies have not observed a positive treatment outcome [36] or the hearing gain was rather small, even though the drug/vehicle persisted in the middle ear for an extended time [23,37]. Other factors such as the cause of hearing loss, amount of hearing loss, timing of treatment, and interaction between the drug and vehicle may have significant effects on the hearing outcome and should be assessed. Second, a third group of animals with no treatment may be required to demonstrate the actual benefit of IT drug delivery in groups 1 and 2. The hearing threshold of the animals with no treatment have been reported in our former study with the same experimental design [23]. However, it is important to compare an untreated ear with a dual vehicle-treated ear in the same animal. Third, we did not measure the drug-transportation efficiency from the middle ear into the inner ear. High-performance liquid chromatography of the perilymphatic fluid can be used to determine the concentration of D inside the cochlea, which will be the subject of a future study.

## 4. Materials and Methods

### 4.1. Experimental Design and Animal Groups

**Figure 6:**
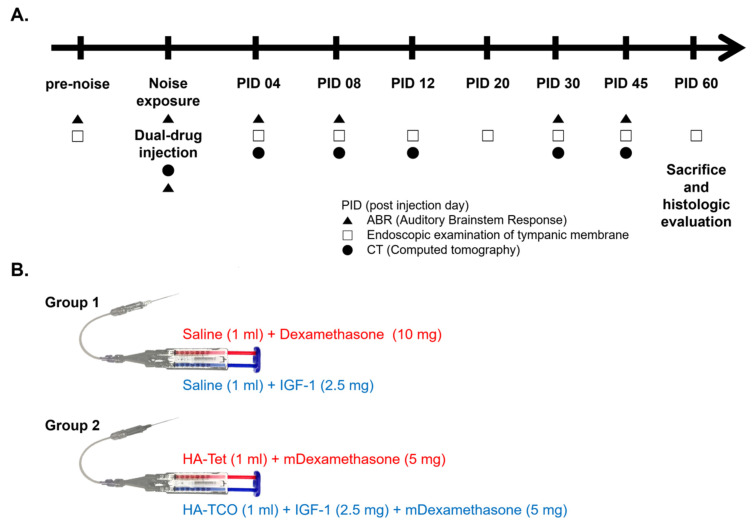
Schematic of experimental design and drug formulation. (**A**) Noise exposure and intra-tympanic injection of dexamethasone and insulin like growth factor 1 (IGF1) were performed on the same day; namely, post injection day (PID) 0. Auditory brainstem response (ABR) measurement, micro CT, and endoscopic examination of the tympanic membrane were performed every 4 to 15 days for 2 months. (**B**) Dexamethasone and IGF1 were used as drugs in groups 1 and 2. Saline and cross-linked HAs were the vehicles in group 1 group 2 respectively. HA-Tet, tetrazine-modified hyaluronic acid; HA-TCO, transcyclooctene-modified HA (HA-TCO).

A total of 28 ears from 16 male Sprague-Dawley (SD) rats (weight: 180–200 g, 6 weeks old) with normal hearing were used to compare treatment outcomes with and without the dual vehicle. A total of 24 ears were randomly assigned to two groups: D and IGF1 in saline (Group 1, n = 13 ears) vs. microcapsulated D (mD) and IGF1 loaded in a cross-linked HA vehicle (Group 2, n = 11 ears). Four ears were excluded from the study due to suboptimal quality of IT injection (poor injection, as described below). D and IGF1 were the active elements (drugs). Cross-linked HA and microcapsules were used as the vehicle. A schematic experimental schedule was drawn to indicate the timing of noise exposure, IT injection of drug/vehicle, ABR, micro CT, and endoscopic examination (Figure 6A). Briefly, the animals were followed for 60 days with ABR, CT, and endoscopy examinations at 4- to 15-day intervals.

In addition, eight ears from four SD rats were used for ICG tracing (Dongindang Pharmaceutical, Siheung, Republic of Korea) to confirm the presence of a drug in the middle ear space. Ears were randomly classified into two groups: ICG (+) ears and ICG (−) ears. The ICG (+) group was injected with 5 mg/mL of ICG in cross-linked HA and the ICG (−) group was injected with a control dye (iodinated dye with no fluorescence) in cross-linked HA. Fluorescence endoscopy was performed at 20- to 30-day intervals as long as the animal survived, up to 129 days.

### 4.2. Animal Model of Acute Acoustic Trauma

A murine model of acute acoustic trauma with hearing loss of 50 to 75 dB SPL was created as described previ ously [38]. Briefly, white noise at 116 dB C was produced with a sine noise generator (Type 1049, Bruel and Kjaer, Naerum, Denmark), power amplifier (MA-620, Inkel, Incheon, Republic of Korea), and speaker (CP800Ti, Beyma, Valencia, Spain). Before noise exposure, rats were anesthetized with a mixture of zoletil (33 mg/kg) and xylazine (8 mg/kg) via intramuscular administration and placed separately within wire-mesh cages. Next, they were exposed to white noise for 3.7 h. A sound-level meter (Type 3604, Yokogawa Electric, Tokyo, Japan) was used to monitor the sound intensity over the acoustic trauma time.

### 4.3. Preparation of Drug/Vehicle Formulations

In total, 10 mg of D (D1961, Tokyo Chemical Industry, Tokyo, Japan) and 2.5 mg of IGF1 (Mecasermin, recombi nant human IGF1, Astellas, Tokyo, Japan) were used as the drugs for both group 1 and group 2. For group 1, normal saline (14200-075, Gibco, Carlsbad, CA, USA) was used as the vehicle. For group 2, cross-linked HA was used as the vehicle and D was loaded in microcapsules.

Biorthogonal click-crosslinking Diels–Alder reaction was used to generate cross-linked HA. Two forms of HA were prepared separately: tetrazine-modified HA (HA-Tet) and transcyclooctene-modified HA (HA-TCO). HA powder (100 mg; Humedix Inc., Anyang, Republic of Korea) was added to separate tubes along with deionized water and 4-(4,6-dimethoxy-1,3,5-triazin-2-yl)-4-methyl-morpholinium chloride. This activated the carboxyl groups in HA. After stirring for 30 min, Tet (3.63 mg, 0.01 mmol; Click Chemistry Tools, Scottsdale, AZ, USA) and TCO (2.63 mg, 0.01 mmol; Click Chemistry Tools) were added to each HA solution. The reaction mixtures were stirred for 24 h and individually dialyzed to remove unreacted Tet and TCO. Following dialysis, the HA-Tet and HA-TCO mixtures were lyophilized in a freeze-dryer (FD8508, Ilshinlab, Daejeon, Korea). When HA-Tet and HA-TCO are blended in the mixing tip of a dual-barrel syringe, a structurally stable cross-linked HA that slowly biodegrades over time is formed. This reaction proceeds rapidly under physiological conditions and has the advantages of facile handing and reproducibility. The degree of bridging is correlated with the degree of covalent bonding between the two types of HA: More covalent bonds increase the strength of the cross-links and increase the biodegradation time. The residence times of cross-linked HA depots are comparatively long. The degree of bridging in group 2 was 1/10,000. For real-time fluorescence endoscopy, the degree of bridging used was much higher (1/250–1/1000) to further extend the residence time of the vehicle.

The microcapsules were spherical particles with a diameter of 50 µm made of PLGA (lactic/glycolic acid = 50/50, MW = 33,000; Birmingham Polymers, Inc., Birmingham, AL, USA). Microcapsules were generated using a mono-axial ultrasonic atomizer. PLGA and D solutions were fed into an ultrasonic atomizer with a mono-axial nozzle. Microdroplets were produced by atomizing the mixed solutions of PLGA and D. Polyvinyl alcohol (Sigma-Aldrich, St. Louis, MO, USA) was used as an emulsifier. The distance between the aqueous polyvinyl alcohol solution and the atomizer head was 1 cm. The resulting solutions were gently stirred to allow for solidification of the microcapsules, followed by filtering and washing with distilled water. The obtained mD was frozen and then freeze-dried. The encapsulation efficiency of Dex was determined using acetonitrile and sodium phosphate.

### 4.4. IT Injection Procedure

The rats were anesthetized with zoletil and xylazine. The drug/vehicle was injected into the middle ear through the tympanic membrane (TM) under a surgical microscope (Opmi Pico, Zeiss, Oberkochen, Germany) at 3 h after termination of noise exposure. In detail, we used a dual-barrel syringe (Kovax-Syringe 1 mL, Korea Vaccine Co., Seoul, Republic of Korea) with a 24 G needle (Angiocath Plus, Becton, Dickinson and Company, Franklin Lakes, NJ, USA) and a mini-extension tube (Mini-Volume Line, Insung Medical, Seoul, Republic of Korea) (Figure 6B). First, an air vent was made in the anterior superior quadrant of the TM and the drug/vehicle was injected into the posterior superior quadrant of the TM. The injection was stopped when the drug/vehicle completely filled the middle ear or it leaked out through the air vent. A total of 30 to 60 μL of drug/vehicle was injected into each ear at a slow speed (~40 µL/10 s). The quality of drug/vehicle injection was classified according to the following three grades: poor, the middle ear cavity was filled with ≤10 µL drug/vehicle; fair, more than half of the middle ear cavity was filled; good, the middle ear cavity was completely filled without air bubbles. Only ears with good or fair injections were included for further analysis. The day of IT drug/vehicle injection was considered day 0 and the post-injection days (PIDs) were counted from this time point.

### 4.5. Micro CT

Images were taken with in vivo micro CT (NFR Polaris-G90, NanoFocusRay, Jeonju, Republic of Korea) to verify the residence time of the drug/vehicle in the middle ear space just after the injection and at PID4, 8, 12, 30, and 45. The presence of drug/vehicle was confirmed when signal attenuation was detected in the middle ear where air was supposed to be found.

### 4.6. Visible Light Endoscopy

The TM was examined to verify the presence of inflammatory adverse reactions or permanent perforation. A 2.7-mm-diameter endoscope (GD-060, Chammed, Gunpo, Republic of Korea) coupled with a smart phone camera (iPhone 4, Apple Inc., Cupertino, CA, USA) was used to photograph the TM pre-injection and at PID4, 8, 12, 20, 30, 45, and 60. Inflammatory adverse reactions were defined as bulging of the pars flaccida or erythematous color change in the TM (Supplementary Figure S1). The inflammatory adverse reaction was considered resolved when the pars flaccida of the TM flattened and the color of the TM returned to normal. TM perforations and healing processes were also observed using the endoscope. Time to complete healing of the TM was calculated based on the endoscopic findings.

### 4.7. Real-Time Fluorescence Endoscopy with ICG

We performed an additional experiment to address the fact that micro CT cannot be used to differentiate the drug from the vehicle based on CT attenuation. ICG was the active element (drug) and cross-linked HA was the vehicle. That is, to evaluate the residence time of the drug independent of the vehicle, ICG was used as a visually trackable drug. Because we did not want the residence time of the vehicle to be an additional variable, we used cross-linked HA with a very high degree of bridging (1/250–1/1000). This allowed the vehicle to persist in the middle ear throughout the experimental period (129 days). Real-time fluorescence endoscopy was performed using the Model-L6k system (Inthesmart, Seoul, Republic of Korea). The excitation wavelength was 808 nm (2.5 to 6.0 W) and the emission wavelength was 825 nm. By overlapping the visible light endoscopic image and near infra-red fluorescence endoscopic image in real time, we were able to visually track the drug behind the TM. The vehicle did not emit any fluorescence, and the intensity of the fluorescence reflected the amount of ICG regardless of the vehicle.

### 4.8. Hearing Threshold Assessment Based on ABR

To measure the hearing threshold, ABRs were measured before noise exposure, at 1 h after noise exposure, and at 3.5 h after drug/vehicle injection on PID4, 8, 30, and 45. Rats were anesthetized as described above. ABRs were measured in a sound-proof chamber using the Smart EP system (Intelligent Hearing Systems, Miami, FL, USA). Subdermal needle electrodes were inserted at the vertex (active electrode) and behind the injected ipsilateral ear (reference electrode) and contralateral ear (ground electrode). The earphone tube was inserted gently into the ear canal. Click auditory stimuli were delivered to the target ear. Hearing thresholds were determined by evaluating the lowest stimulus level for waves III/V and SN_10_ (slow, negative wave) recognition from 90 dB SPL with a 5-dB SPL decrease.

### 4.9. Inner Ear Hair Cell Count

Rats were sacrificed on PID60, and bullae were harvested for histopathologic examination. Each specimen was fixed with 4% paraformaldehyde for 12 h at 4 °C and rinsed three times with phosphate buffered saline for 5 min. The cochlea was separated from the bulla and decalcified in 10% ethylenediamine tetraacetic acid solution for 4 weeks. Membranous cochlea was separated from bony cochlea in a petri dish under a stereoscopic microscope (DSZ-40T, Dongwon, Bucheon, Republic of Korea). The cochlea was stained with a mixture of phalloidin and Triton X100 (1:100) and rinsed once more in the same manner. The prepared membranous cochlea was separated into three parts (apical, middle, and basal turns corresponding to 8, 16, and 32 kHz, respectively) and mounted on slides. Z-stack images were taken using a confocal microscope (TCS SP8, Leica Microsystems, Wetzlar, Germany), and hair cells were counted over a 200- µm distance in each turn. 

### 4.10. Middle Ear Histology of the Drug/Vehicle after Contacting the Mucosa

On the last day of hearing evaluation (PID60), the bulla was fixed, rinsed, and decalcified as described above. The bulla bone was embedded in paraffin wax and cut into 5-μm-thick sections. Sections were stained with hematoxylin and eosin. The TM and mucosa at the base of the bulla were observed with a light microscope (CX31, Olympus, Tokyo, Japan). The consistent location of the TM was determined by identifying the section in which the malleus head and its fibrous connection to the TM could be observed. The thickness of the TM and bulla mucosa was measured using DP2-BSW software (Olympus).

### 4.11. Statistical Analysis

The mean differences in ABR threshold, hair cell count, time to complete healing of the TM, residence time of drug in the middle ear space, thickness of mucosa, and incidence of inflammation were compared between the two groups using the Mann-Whitney *U* test and Fisher’s exact test. The differences in ABR threshold over time in each group were compared using the Wilcoxon signed rank test with IBM SPSS version 19.0 (IBM Co., Armonk, NY, USA). Data are presented as the means ± standard errors, and differences were considered statistically significant at *p* < 0.05.

## 5. Conclusions

The residence time of the drug/vehicle in the middle ear was extended 10.9 times using the dual vehicle; namely, cross-linked HA and PLGA microcapsules. Considering that the cross-linked HA functioned as a drug depot and released ICG for 2 to 3 months, the dual vehicle may sustain and slowly release the drug. The treatment outcome in terms of hearing threshold and hair cell count was comparably good in both groups treated with IT D and IGF1. Unfortunately, the IT procedure and/or the dual vehicle was associated with inflammatory adverse effects in one-third of the cases. However, these adverse effects were transient and non-significant after 2 months.

## Figures and Tables

**Table 1:** The number of hair cells in the basal, middle, and apical turns of the cochlea OHC, outer hair.

Number of Hair Cells (/200 μm)	OHCs in Basal Turn	OHCs in Middle Turn	OHCs in Apical Turn	IHCs in All Three Turns
Group 1 (n = 9)	73 ± 0.7	73.1 ± 0.7	73.7 ± 0.6	54.7 ± 0.8
Group 2 (n = 8)	72 ± 1.4	71.6 ± 0.8	73.6 ± 1.1	55.3 ± 0.9
*p* value	0.743 (ns)	0.093 (ns)	0.888 (ns)	0.864 (ns)

The mean differences in the number of hair cells in each region were compared between the group 1 and group 2 using the Mann-Whitney *U* test. ns: not significant cell; IHC, inner hair cell.

## Data Availability

All data generated or analysed in this study are included in this published article.

## Supplementary Materials

The following are available online at https://www.mdpi.com/article/10.3390/ijms22115720/s1.

## Institutional Animal Care and Use Committee Statement

All procedures in the study were approved by the Institutional Animal Care and Use Committee of Seoul National University Hospital (18-0046-C1A0(3)).
