# Supplementary material for: Effect and Biocompatibility of a Cross-Linked Hyaluronic Acid and Polylactide-co-glycolide Microcapsule Vehicle in Intratympanic Drug Delivery for Treating Acute Acoustic Trauma
Source: Int J Mol Sci. 2021 May 27;22(11):5720. doi: 10.3390/ijms22115720 (PMC8198354; doi:10.3390/ijms22115720)
Supplement: Supplementary file 1 [file ijms-22-05720-s001.zip › ijms-1242891-supplementary.pdf]

Supplementary Figure legends

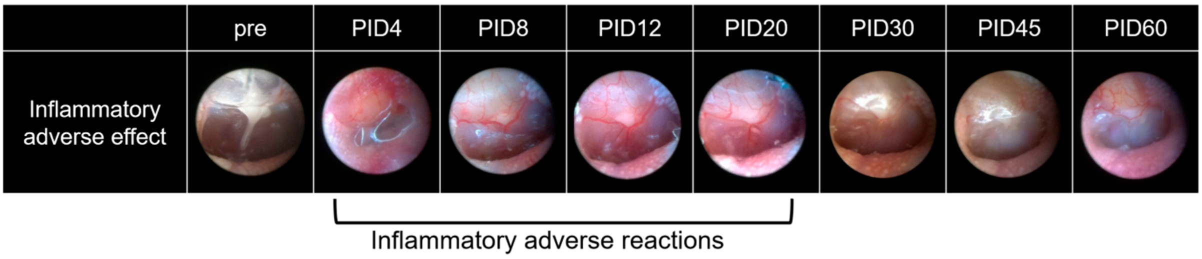

Figure S1. Typical endoscopy findings indicating inflammatory adverse reactions after dual-vehicle injection. Bulging of the pars flaccida and erythematous change of the tympanic membrane were identified between post-injection day (PID) 4 and 20, which slowly recovered after PID30.

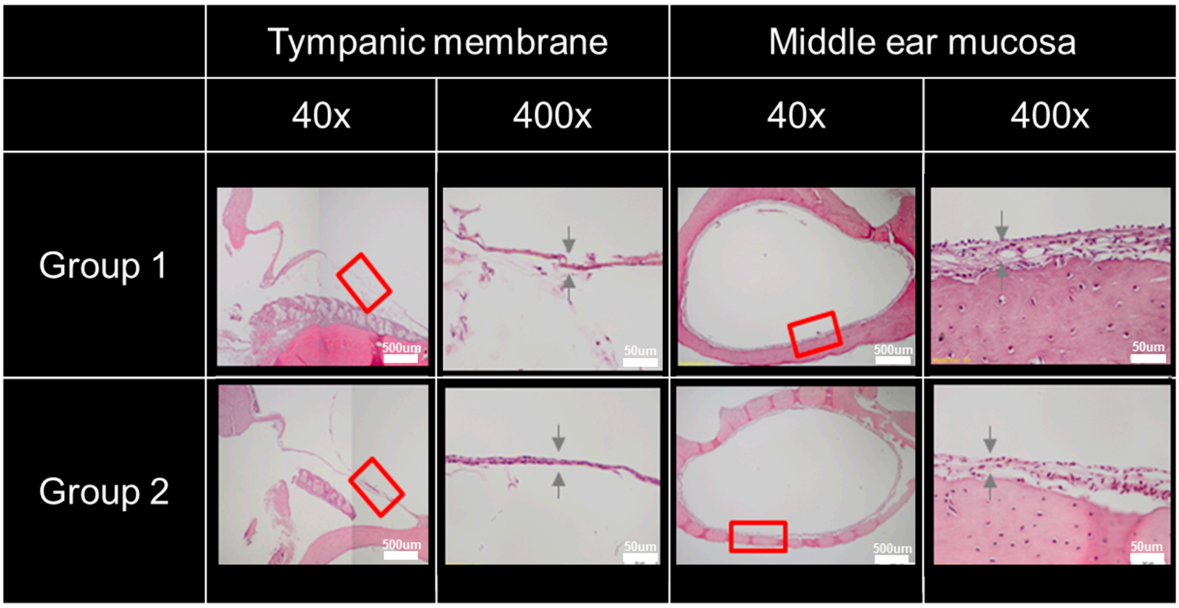

Figure S2. Histopathology of the tympanic membrane (TM) and middle ear mucosa. There were no differences in the TM and mucosa at the base of the bulla between groups 1 and 2. The thickness of the TM was 12.4 ± 14.9 µm in group 1 and 13.3 ± 12.8 µm in group 2. The thickness of the mucosa at the base of the bulla was 22.3 ± 7.3 µm in group 1 and 24.0 ± 16.0 µm in group 2.
